# Alcohol Use Associated With Gambling Harm in a Population Representative Australian Sample

**DOI:** 10.1111/dar.70164

**Published:** 2026-05-04

**Authors:** Koen Smit, Heng Jiang, Robin Room, Aino Suomi, Markus H. Hahn, Sarah MacLean, Anne‐Marie Laslett

**Affiliations:** ^1^ Centre for Alcohol Policy Research, School of Psychology and Public Health La Trobe University Melbourne Australia; ^2^ Melbourne School of Population and Global Health The University of Melbourne Melbourne Australia; ^3^ Department of Public Health, School of Psychology and Public Health La Trobe University Melbourne Australia; ^4^ Centre for Social Research on Alcohol and Drugs, Department of Public Health Sciences Stockholm University Stockholm Sweden; ^5^ Centre for Gambling Research Australian National University Canberra Australia; ^6^ POLIS: The Centre for Social Policy Research Australian National University Canberra Australia; ^7^ Social Work and Social Policy, School of Allied Health, Human Services and Sport La Trobe University Melbourne Australia

**Keywords:** alcohol use, alcohol use while gambling, financial harm, gambling harm, heavy episodic drinking, public health

## Abstract

**Introduction:**

This study examined whether alcohol use while gambling and engaging in heavy episodic drinking (HED) are associated with gambling harm in Victoria, Australia. It also explored whether combined alcohol use behaviours further increase harm, and whether economically disadvantaged individuals experience greater harm from alcohol use.

**Methods:**

Data were used from the representative 2023 Victorian Population Gambling and Health Study subsample (*N* = 3114; 47.9% female; mean age = 46). Regressions were used to test whether HED (six or more drinks per occasion) and drinking while gambling predicted gambling harm and number of harms, measured using the Short Gambling Harm Screen.

**Results:**

Alcohol use while gambling was associated with gambling harm (OR = 2.58, 95% CI [1.53, 4.35], *p* < 0.001) and number of harms (IRR = 2.57, 95% CI [1.65, 4.00], *p* < 0.001), controlling for HED, gambling expenditure and socio‐demographic variables. HED was associated with harm in bivariable models only (OR = 2.20, *p* < 0.001), not in adjusted models. The interaction between HED and consuming alcohol while gambling was associated with increased gambling harm.

**Discussion and Conclusions:**

Drinking while gambling was associated with increased gambling harm, as were combined alcohol use behaviours. HED was not independently associated with harm. Those with heavier drinking patterns who also consumed alcohol while gambling were at particularly elevated risk. Findings are consistent with restricting alcohol use in gambling venues to reduce gambling‐related harm.

## 1 Introduction

In 2023, 61% of Australians engaged in gambling [1], with about 12% of the population reporting gambling‐related harm. In Victoria, just over half of the population (53.3%) engaged in gambling in 2023 [2]. Despite steadily decreasing participation rates over the past 15 years, high‐risk gambling and gambling harm remained stable, with 13% of Victorians who gambled reporting at least one form of gambling harm (compared to 9.6% in 2018–2019). Gambling harms refer to any adverse consequences of gambling, such as mental health problems, financial insecurity (e.g., loss of savings) and problems in social relationships [1, 3, 4]. Although gambling is often accompanied by alcohol consumption, which may influence judgement and decision making, experimental evidence on this effect is mixed [5]. Other studies indicated that higher rates of alcohol consumption are associated with both increased gambling participation [6] and gambling expenditure [7]. However, few studies examined whether alcohol use is associated with gambling harm specifically, rather than high‐risk gambling.

There is increased recognition that gambling harms extend beyond clinical high‐risk gambling. Gambling harms are defined as adverse consequences due to engagement with gambling, leading to lower health or wellbeing [8, 9, 10]. Gambling causes harm to individuals, families and communities. These harms manifest in various domains, such as financial problems [11], legal issues [12], relational harm through increased child neglect, conflicts and family dysfunction including domestic violence [13, 14, 15, 16, 17] and psychological and emotional harm, for example through financial loss [18].

Similarly, harmful alcohol use is associated with substantial health [19, 20] and social harms such as injury, financial stress and harms to others [21, 22, 23, 24, 25, 26]. As gambling and alcohol use often co‐occur and are both associated with higher risks of various harms, the question remains whether alcohol use is associated with gambling‐related harm.

Alcohol and gambling behaviours are connected in various ways [27] as they are often accessible in the same physical environments, including clubs, pubs, hotels and casinos. Gambling providers such as casinos may recognise that alcohol affects self‐control [28] and increases risk‐taking behaviour [29]. In addition, alcohol use and gambling can both lead to behavioural dependency with a significant overlap in underlying risk factors and psychological profiles related to control inhibition and impulsivity [30, 31]. These psychological features are evident in both clinical and non‐clinical samples [32]. Given these shared environmental and psychological features, it is likely that alcohol use is associated with gambling‐related harms, although more research is needed to confirm this dynamic.

Studies focusing on the association between alcohol use and specific gambling‐related harms are scarce. Although various studies show that alcohol can have psychological and expectancy‐based effects on risk‐taking during gambling [28, 33], a recent systematic review of experimental studies concluded that alcohol does not consistently increase gambling‐specific risk‐taking behaviour [5]. Evidence from observational studies, however, indicated that alcohol consumption is associated with greater gambling expenditure and faster loss of available funds [29, 33, 34]. Our previous study, using data from the 2018–2019 Victorian Population Gambling and Health Study, found that heavy episodic drinking (HED) and alcohol use while gambling were associated with increased odds of risky gambling as measured by the Problem Gambling Severity Index (PGSI) [6]. The PGSI is an instrument designed to classify high‐risk gambling severity, capturing features of addiction alongside some common harms [35]. While essential for estimating prevalence of high‐risk gambling, such measures are not designed to identify harms experienced across the broader population. In contrast, gambling‐related harm refers to realised adverse consequences affecting health and wellbeing at individual, family and community levels [8, 9]. Although related, gambling risk and gambling‐related harm are overlapping but distinct constructs. The association between alcohol use and the actual negative consequences of gambling therefore remains less clear.

The current study examined whether alcohol use while gambling and/or HED are associated with any gambling harm and the number of gambling harms experienced by those who gambled in the last year. As direct measurement of HED within gambling settings was not available, general HED frequency is used as an indicator of likely heavy drinking within gambling contexts. Both HED and alcohol use while gambling may independently impair decision‐making and self‐control, and their combination may amplify gambling‐related harm. It was therefore expected that both measures of alcohol use (HED and alcohol use while gambling) would be associated with higher odds and number of gambling‐related harms. Secondly, we examined whether HED and alcohol use while gambling interacted in predicting gambling‐related harm, with the expectation that those who drink more heavily overall and more often while gambling might be substantially more affected by alcohol. As economically disadvantaged people are disproportionately affected in terms of harms from a given level of alcohol use [25, 36] and from gambling [37], a secondary aim of this paper was to investigate whether socio‐demographic backgrounds were associated with occurrence and levels of gambling‐related harms in Victoria, Australia.

## 2 Methods

### 2.1 Study Design and Procedure

Data were derived from the 2023 Victorian Population Gambling and Health Study, a mobile‐phone‐only survey collected between January and May 2023 by the Social Research Centre. The questionnaire was designed by the Australian National University Centre for Social Research Methods. The study acquired a representative sample of residents of the Australian state of Victoria. Detailed information about procedures can be found elsewhere [2].

A total of 11,000 respondents completed the survey. Although most respondents completed a shorter questionnaire, a random subsample of 3156 adults was asked additional questions, including their alcohol use patterns. This resulted in a subsample of 3114 Victorians (47.9% female, M_age_ = 46 years, SD = 18.3, range:18–93) which was used in the current analysis.

The study followed the Strengthening the Reporting of Observational Studies in Epidemiology (STROBE) guidelines [38]. The study used de‐identified secondary data, and no direct patient or public involvement was feasible or required. Ethics approval for data collection and secondary data analysis was obtained from the ethics committees at the Australian National University (protocol number 2022/457) and La Trobe University (HEC24068).

### 2.2 Measures

#### 2.2.1 Gambling Harms Score

Gambling harm was assessed using an expanded version of The Short Gambling Harm Screen (GHS‐10) developed to assess the degree and type of harms caused by gambling [39]. The survey included 10 items on financial, psychological and interpersonal harm. This scale has demonstrated excellent reliability (*α* = 0.98) and validity in Australian population surveys [39, 40]. Four items were added because of their policy relevance to Victoria [10, 40]. Examples included “Been a victim of family or domestic violence” and “Didn't attend fully to the needs of children.” The expanded scale retained a high level of internal consistency (*α* = 0.87 with the four additional items vs. *α* = 0.86 without), indicating no evidence of distortion. For logistic regressions we created a binary score, with 0 (no harms indicated) and 1 (one or more harm indicated). In addition, we created a summary score of all harm items (range 0–13). See Table S1 for individual items and the prevalence of each specific gambling harm.

#### 2.2.2 Alcohol Use

##### 2.2.2.1 Frequency of Alcohol Use While Gambling

Participants responded to “During the past 12 months, how often did you drink alcohol whilst gambling?” on a 5‐point Likert scale with the options 1 (never), 2 (rarely), 3 (sometimes), 4 (often) and 5 (always). Those who did not know or refused to answer were coded as missing (*n* = 4, 0.2%). Responses were recoded into three categories: Never, Sometimes/rarely and Often/always.

##### 2.2.2.2 Heavy Episodic Drinking

The third item of the Alcohol Use Disorders Identification Test (AUDIT‐C) was used to examine HED [41]. For our analysis, the general frequency of consuming six or more drinks (i.e., 60 g of pure alcohol) on an occasion in the previous 12 months was transformed into: never, less than monthly, monthly, weekly or daily (HED).

#### 2.2.3 Covariates

##### 2.2.3.1 Gambling Participation

Gambling activity was documented as a binary outcome and assessed as either any gambling (1) or no gambling (0) in the previous 12 months.

##### 2.2.3.2 Gambling Frequency

Participants indicated how often they engaged in various gambling activities (e.g., lottery, sports betting, pokies and online gambling) over a specified period. A respondent's overall frequency is the sum of all gambling activities, expressed as the number of activities *per year*. These were categorised into four yearly frequency groups: Never (0 times), Low (1–11 times), Medium (12–51 times) and High (52+ times).

##### 2.2.3.3 Gambling Expenditure

Yearly expenditure on various gambling products was dichotomised following previous research [2]. Our variable measures heavy gambling expenditure, categorising gambling spending into 0 (spending < A$5000) and 1 (spending A$5000 or more).

##### 2.2.3.4 Demographics

Gender, age group, region (Melbourne/outside Melbourne), ethnicity (indicated by whether English or another language is used at home) and annual personal income were recorded (see Table 1 for details).

**TABLE 1:** Descriptive statistics by totals and by gambling harm (no harm vs. any harm).

	Total (3150)		Of those who gambled in the last year (1918)		
	*N*	%	% No harm	One or more harms	Number of harms (mean and SE)
Gender					
Male	1622	48.2%	80.4%	19.6%	0.54 (0.05)
Female	1492	50.9	92.0%	8.0%	0.18 (0.02)
Other	30^a^	0.01	87.4%	13.7%	0.55 (0.44)
Age, years					
18–34	1008	30.5%	79.9%	10.1%	0.59 (0.07)
35–49	710	24.0	80.3%	19.7%	0.51 (0.07)
50–64	743	24.2	88.5%	11.5%	0.28 (0.05)
65+	579	21.4	92.7%	7.3%	0.12 (0.03)
Region in Victoria					
Melbourne	2411	75.6%	84.0%	16.0%	0.42 (0.04)
Regional	733	24.4	90.2%	9.8%	0.23 (0.04)
First language at home					
English	2318	70.4%	88.2%	11.8%	0.29 (0.02)
Other	821	29.6	77.6%	22.4%	0.63 (0.09)
Personal income, per year					
Nil—$41,599	643	20.0%	85.4%	14.6%	0.45 (0.07)
$41,600—$77,999	555	16.7	84.0%	16.0%	0.40 (0.07)
$78,000 or more	829	22.7	85.3%	14.7%	0.38 (0.05)
Missing	1123	40.5	87.2%	12.8%	0.29 (0.04)
Gambling frequency					
Never	1238	47.6%	—	—	—
Low	800	25.3	91.5%	8.5%	0.19 (0.03)
Medium	461	11.8	82.6%	17.4%	0.34 (0.05)
High	571	15.3	81.3%	18.7%	0.59 (0.07)
Gambling expenditure					
< $A5000	1633	96.2%	88.9%	11.1%	0.20 (0.02)
≥ $A500	171	3.8	27.2%	72.8%	3.50 (0.38)
HED					
Never	1796	63.1%	87.6%	12.4%	0.30 (0.04)
Less than monthly	759	23.1	85.7%	14.3%	0.33 (0.04)
Monthly	350	8.7	83.6%	16.4%	0.36 (0.07)
Weekly or daily	211	5.1	76.3%	23.7%	1.00 (0.19)
Drinking alcohol while gambling^a^					
Never	1223	72.2%	89.4%	10.6%	0.22 (0.02)
Sometimes	411	18.8	80.6%	19.4%	0.62 (0.09)
Often/always	268	0.9	68.8%	31.2%	0.95 (0.15)

*Note:* (Unweighted Ns, weighted %s).

Abbreviation: HED, heavy episodic drinking.

^a^ Excluded from subsample analysis due to low *N*.

### 2.3 Analyses

We conducted descriptive analyses and then logistic regression to test the association between alcohol use while gambling and heavy episodic drinking (HED: consuming six or more drinks on one occasion) with any gambling harm (none vs. one or more harms) amongst those who indicated having gambled in the last year. Subsequent analyses were conducted in two stages: (i) a logistic regression for the association of alcohol use with both any gambling harm (none vs. one or more harms); and (ii) a negative binomial regression for the association of alcohol use with the number of harms. A hurdle approach allows for separate evaluation of the likelihood of experiencing any gambling‐related harm (any harm vs. no harm) and the severity of harm (count). The negative binomial model was chosen due to the skewed distribution of the total number of harms. Incidence rate ratios (IRR) were reported, with IRR > 1.00 indicating a higher count of harms. We also tested an interaction term between alcohol while gambling and HED in both the logistic and negative binomial models to examine whether combined drinking patterns predicted greater gambling harm. Both alcohol use while gambling and HED were entered simultaneously as predictors in all multivariable models. Multivariable models controlled for gambling frequency, gambling expenditure (≥ $5 k) and socio‐demographic covariates (e.g., gender, age, region, language at home and income). As a robustness cheque, all analyses were repeated using the validated 10‐item Short Gambling Harm Scale. Results remained the same (not shown).

Missing income responses (*n* = 1123) were retained as a separate “missing” category to maximise sample retention, whereas missing responses for alcohol‐use variables (*n* = 4) were excluded from analyses. As overall missingness was low, no imputation procedures were applied. Multicollinearity diagnostics were based on variance inflation factors (VIF), using a threshold of VIF ≥ 4 to indicate problematic collinearity [42]. Weighted data, with the total N set to the sample size, were used to adjust the findings to the Victorian population [10]. All analyses were performed using Stata 17 [43].

## 3 Results

### 3.1 Characteristics of the Sample

Sample characteristics, including gambling prevalence estimates by socio‐demographic characteristics for the full population and for those who gambled during the last year, are reported in Table 1. Amongst those who gambled (*N* = 1918), after applying population weights that account for sampling biases, we found that about 14% experienced any form of gambling harm (Table 1). Specifically, 5.7% indicated one harm, whereas 8.1% indicated two or more harms. More men than women reported gambling harms (19.6% vs. 8%). Younger age groups were more likely to report gambling harms (between 10% and 19.5%). Contrastingly, a larger majority of those aged 65+ (92.7%) reported no gambling harm. Regional Victorians and those who speak English at home reported relatively lower rates of any *gambling harms*. Those who reported a higher gambling frequency and gambling expenditure reported a relatively higher number of gambling harms. About 73% of those spending A$5000 or more on gambling yearly reported two or more gambling harms. Notably, those who reported both alcohol use while gambling and HED more frequently reported a higher number of gambling harms.

### 3.2 Association of Alcohol Use and Any Harm

Logistic regression results show that alcohol use while gambling is strongly associated with any gambling harm (Table 2). Individuals who sometimes drank while gambling had two times higher odds of reporting any harm, whereas those who often or always used alcohol while gambling had two to four times higher odds. In the bivariable model, those engaging in HED at least weekly were 2.2 times more likely to report harm from gambling than those who never drank heavily, while those who reported HED less frequently did not exhibit higher odds of gambling harm. The association with HED disappeared when alcohol while gambling and covariates (i.e., socio‐demographics and gambling indicators) were considered in the same model.

**TABLE 2:** Unadjusted and adjusted odds of any gambling harm (0 vs. 1+ harms indicated) by alcohol use, heavy episodic drinking (HED) and socio‐demographic characteristics (Main Effects Model).

Any gambling harm	Bivariable	Any harm—multivariable				Number of harms—multivariable			
	Unadjusted OR	Adjusted OR	95% CI		*p*	Adjusted IRR	95% CI		*p*
Alcohol while gambling									
Never	1.00	1.00	—	—	—	1.00	—	—	—
Sometimes	2.03***	1.67**	1.10	2.53	0.017	2.45***	1.68	3.57	< 0.001
Often/always	3.83***	2.58***	1.54	4.34	< 0.001	2.57***	1.65	4.00	< 0.001
HED									
Never in last year	1.00	1.00	—	—	—	1.00	—	—	—
Less than monthly	1.18	0.76	0.51	1.14	0.196	0.66*	0.44	0.96	0.032
Monthly	1.39	0.77	0.46	1.28	0.311	0.66	0.40	1.07	0.088
Weekly or daily	2.20***	0.65	0.35	1.21	0.171	0.59	0.34	1.01	0.056
Gambling frequency									
Low	1.00	1.00	—	—	—	1.00	—	—	—
Medium	2.26***	2.13**	1.39	3.27	0.001	1.49	0.98	2.28	0.064
High	2.46***	2.10**	1.30	3.39	0.002	1.35	0.83	2.19	0.228
Gambling expenditure									
< $A5000	1.00					1.00	—	—	—
≥ $A5000	21.39***	13.59***	7.07	26.13	< 0.001	13.58***	8.73	21.13	< 0.001
Gender^a^									
Male	1.00	1.00	—	—	—	1.00	—	—	—
Female	0.38***	0.57**	0.40	0.80	0.001	0.68*	0.49	0.95	0.025
Age									
18–34	1.00	1.00	—	—	—	1.00	—	—	—
35–49	0.98	1.18	0.76	1.81	0.459	0.84	0.56	1.26	0.394
50–64	0.52***	0.56*	0.36	0.789	0.013	0.48**	0.31	0.75	0.001
65+	0.31***	0.32**	0.17	0.61	0.001	0.24***	0.12	0.48	< 0.001
Region in Victoria									
Melbourne	1.00	1.00	—	—	—	1.00	—	—	—
Regional	0.57**	0.61*	0.40	0.94	0.024	0.48***	0.33	0.69	0.000
First language at home									
English	1.00	1.00	—	—	—	1.00	—	—	—
Other	2.15***	1.69*	1.10	2.62	0.017	1.27	0.87	1.85	0.224
Personal Income, per year									
Nil—$41,599	1.00	1.00	—	—	—	1.00	—	—	—
$41,600–$77,999	1.12	0.87	0.52	1.45	0.595	0.58*	0.36	0.93	0.024
$78,000 or more	1.01	0.50*	0.30	0.83	0.007	0.46**	0.29	0.74	0.001
Missing	0.86	0.78	0.47	1.31	0.347	0.74	0.44	1.25	0.262

*Note:* *p < 0.05, **p < 0.01, ***p < 0.001; Exact *p*‐values are provided for the multivariable analyses.

Abbreviations: CI, confidence interval; IRR, incidence risk ratio; OR, odds ratio.

^a^ The “non‐binary or other” category is excluded from subsample analysis due to low *N*.

Those who reported a medium and high gambling frequency had two times higher odds of reporting any gambling harm. Higher odds for harm were found amongst those spending ≥ A$5000 on gambling per year (odds ratio [OR] = 13.39, *p* < 0.001). In addition, the bivariable and multivariable models showed that women, those aged 50+, and those in regional areas had the lowest odds of reporting any gambling harm. Conversely, those living in homes where English was not the first language had around twice the odds of reporting any gambling harm. Income appeared unrelated to gambling harms in these models. Importantly, even when controlling for gambling expenditure and frequency and socio‐demographic variables, the association of alcohol use while gambling with any gambling harm remained. Sensitivity analyses without weights yielded similar results (results not shown).

### 3.3 Association of Alcohol Use and Number of Gambling Harms

Given that the overdispersion parameter *α* is significantly greater than zero (7.77; 95% confidence interval 6.29–9.60), a negative binomial model was more appropriate than Poisson when we modelled the number of gambling harms. Results (Table 2) indicate that individuals who sometimes drank while gambling reported approximately 2.4 times higher rates of gambling harm than those who never drank while gambling (IRR = 2.43, *p* < 0.001), and those who often/always drank while gambling reported 2.5 times higher rates. In contrast, none of the HED categories remained significantly associated with the number of harms in the fully adjusted multivariable model.

Those who spent ≥ A$5000 per year on gambling reported substantially higher harm rates. Women had lower rates of harm than men, and older age groups, regional residents and those with higher income also reported fewer harms. Using a language other than English at home did not significantly increase the rate of gambling harms once covariates were included. Consistent with the logistic model, the association between alcohol use while gambling and gambling harm persisted even after controlling for gambling indicators and socio‐demographic variables. Sensitivity analyses without weights yielded similar results (results not shown).

### 3.4 Interaction of Alcohol While Gambling and HED

**FIGURE 1:**
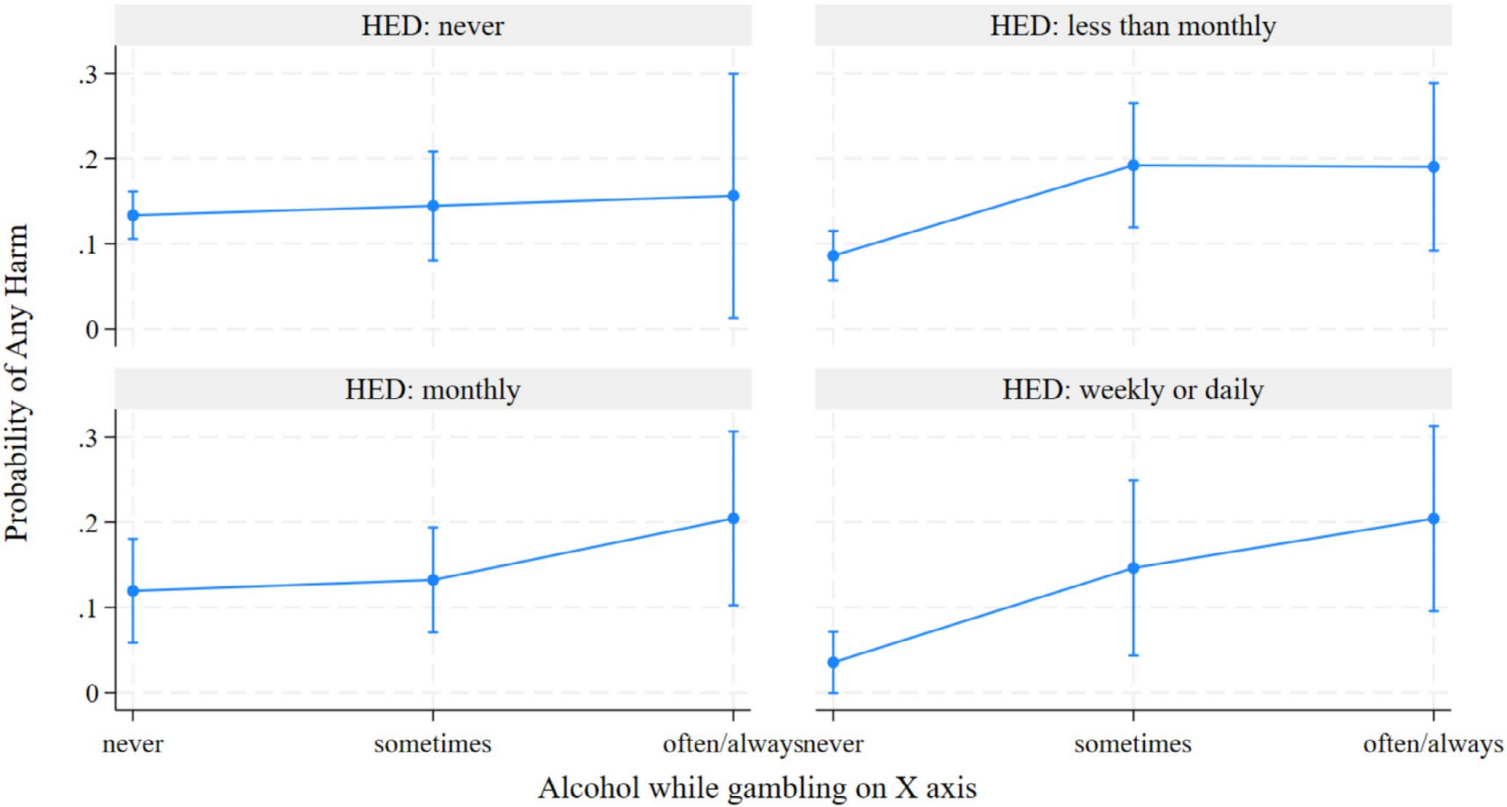
Probability of gambling harm by interaction between alcohol while gambling and heavy episodic drinking (HED): An increased probability of any gambling harm when both occur frequently (controlling for covariates).

The interaction of alcohol use while gambling and HED with any versus no gambling harm was significant (Table 3). Specifically, individuals who often or always drink while gambling and engaged in HED on a weekly or daily basis have increased odds of experiencing any gambling harm (OR = 8.02, *p* = 0.036) while controlling for gambling frequency, gambling expenditure (≥ 5 k) and socio‐demographic variables. Furthermore, in the negative binomial model for the number of harms (right side of Table 3), this same group showed a significantly higher rate of harm (IRR = 12.63, *p* = 0.008). Figure 1 illustrates the increased probability of gambling‐related harms by levels of alcohol use while gambling and HED.

**TABLE 3:** Interaction effects of alcohol use while gambling (AWG) and heavy episodic drinking (HED) on the odds of any gambling harm (1 or 2+ harms indicated).

Interaction AWG × HED	Any harm (0 vs. 1+)				Number of harms			
	Adjusted OR	95% CI		*p*	IRR	95% CI		*p*
Never × Never	1.00	—	—	—	1.00	—	—	—
Sometimes × Less than monthly	2.72*	**1.01**	**7.31**	**0.048**	3.41**	**1.45**	**7.86**	**0.004**
Sometimes × Monthly	1.03	0.31	3.38	0.892	1.03	0.37	3.10	0.888
Sometimes × Weekly or daily	5.54*	**1.03**	**29.78**	**0.046**	8.02**	**1.62**	**39.54**	**0.011**
Often/always × Less than monthly	3.41	0.43	8.67	0.283	1.97	0.45	8.67	0.372
Often/always × Monthly	1.72	0.32	9.17	0.528	1.86	0.41	8.45	0.420
Often/always × Weekly or daily	8.02*	**1.14**	**56.40**	**0.036**	12.63**	**1.93**	**82.55**	**0.008**

*Note:* Exact *p*‐values are provided for the multivariable analyses. Bold values indicate statistically significant associations (*p* < 0.05).

Abbreviations: CI, confidence interval; IRR, incidence risk ratio; OR, odds ratio.

^*^ *p* < 0.05.

^**^ *p* < 0.01.

Sensitivity analyses showed that weighting influenced the interaction effects for both the logistic and negative binomial regressions (see Table S2). For example, the interaction between frequent alcohol use while gambling and weekly/daily HED was statistically significant in the weighted logistic regression (OR = 8.02) but was non‐significant when unweighted (OR = 3.39, *p* = 0.137). For the negative binomial regression, the interactions also weakened, although the results for the combination of frequent alcohol while gambling and weekly/daily HED remained the same (IRR = 6.20, *p* = 0.026). Although the non‐significant effects were still in the same direction, the differences suggest that survey weighting influenced the observed interaction terms somewhat.

## 4 Discussion

This study indicated that both alcohol use while gambling and HED were associated with higher odds of experiencing gambling‐related harm and with a higher number of gambling harms. However, when controlling for covariates, the associations of HED with any gambling harm and number of harms were no longer statistically significant. Interestingly, we found an interaction where both engaging in alcohol use while gambling and reporting a higher frequency of HED were associated with increased risk of experiencing gambling harm.

The findings align with previous literature on the relationship between alcohol use and risky gambling which shows that alcohol use exacerbates risk‐taking while gambling [33]. A systematic review concluded that an association between alcohol use and risk‐taking behaviour while gambling was not clearly demonstrated in lab tasks [5]. Our studies, however, show that alcohol use while gambling exacerbates risky gambling [6]. Unlike our previous analysis [6] focusing on gambling risk (PGSI), the present study examined gambling‐related harms as a distinct public health outcome. This distinction is important, as elevated gambling risk does not necessarily translate into realised harm. The analyses demonstrated that this was the case for both any harm, as well as the number of harms experienced, even when considering gambling expenditure and gambling frequency. One potential explanation is that the cost of alcohol use while gambling adds to the existing (financial) burden of gambling. Another explanation is that inebriated individuals may make gambling‐related decisions that they later regret, financially or otherwise. Conversely, the association of HED and any harm disappeared while controlling for alcohol while gambling and other factors, suggesting that HED alone does not independently explain gambling harm once other factors are accounted for. This may be because harm arises primarily from alcohol use within the gambling environment, rather than from drinking more heavily in general.

Interaction analyses suggest that frequent heavy drinkers who also consume alcohol in gambling contexts are particularly vulnerable to harm. Although weighting affected the strength of some of the interaction terms, effects remained consistent. This likely reflects the low prevalence of combined exposure groups. The direction of effects remained consistent across weighted and unweighted models. However, the findings should be considered preliminary pending replication in larger samples. Although we cannot determine how much general HED occurred specifically within gambling contexts, the moderate correlation between HED and alcohol use while gambling (*r* = 0.50, *p* < 0.001) and prior qualitative evidence suggest that a substantial portion of heavy drinking occurs in gambling settings [44]. We therefore treated general HED as a proxy for heavy drinking while gambling, acknowledging that this assumption introduces some measurement imprecision in estimating HED's independent effects. Importantly, future research should explore the mechanisms behind this interaction.

Observed socio‐demographic differences in gambling harm provide insight into the populations at risk for gambling harm. Women, older individuals (50+ years old) and those residing outside metropolitan Melbourne were less likely to report gambling‐related harm and had lower counts of harm. Conversely, speaking a language other than English at home was associated with any gambling‐related harm, but not with number of harms. In contrast with previous research [37], we found that higher incomes were associated with lower risk for any gambling harms as well as a lower number of harms. Simultaneously, we found that higher expenditure on gambling (> A$5000) was associated with higher risk for any gambling harm and a higher number of harms, and that a higher gambling frequency was associated with any harm, but not with the number of harms. Altogether, these findings highlight specific socio‐demographic groups and behaviours (e.g., frequent gambling and higher gambling expenditure) as important targets for harm‐reduction.

### 4.1 Limitations and Future Research

Strengths include use of a large population‐based Victorian sample with weighting, and use of validated harm measures (Short Gambling Harm Scale) capturing harms beyond gambling risk. However, several limitations need to be acknowledged. First, although results are consistent between any gambling harm and the number of gambling harms, this study combined analyses of various gambling product types which may exaggerate or mask specific problems. Additionally, HED was measured as a general drinking pattern rather than specifically within gambling contexts, which may introduce measurement error in our estimates of HED's independent effects. It should also be acknowledged that all measures were self‐reported and may be subject to recall and social desirability biases. Second, a limitation in our analytical approach is that we included binary or categorical covariates. For example, harms associated with spending more than A$5000 may differ substantially between lower‐ and higher income earners. Third, at the time of data collection, the state of Victoria had just come out of COVID‐19 lockdowns, and COVID‐19 disruptions may have influenced gambling behaviour (e.g., more online gambling [2]), and on‐ and off‐premises alcohol use. Fourth, although results were in a similar direction, sensitivity analyses revealed that the interaction weakened when population weights were removed. This suggests that the interaction is sensitive to sample weighting and highlights the need for cautious interpretation of the results. Lastly, the study used a cross‐sectional dataset. As such, we can only discuss associations, and longitudinal studies are crucial to further understanding. Furthermore, residual confounding is possible, including individual differences (e.g., impulsivity, risk‐taking or comorbid substance use) that may predispose individuals to both drinking while gambling and experiencing gambling harm.

### 4.2 Implications for Public Health

Because drinking while gambling was independently associated with harm, venue‐level strategies that limit alcohol availability or service, such as stricter controls on purchase by intoxicated patrons, reduced drink promotions or separation of bar and gambling areas, may reduce gambling‐related harm. These factors are modifiable and operate independently of gamblers' socio‐demographic risk profiles. Findings are consistent with the potential value of restricting alcohol availability during gambling as a harm‐reduction strategy; however, causal inference cannot be made from these cross‐sectional data. The findings further suggest that prevention and intervention should focus on specific socio‐demographic groups who are vulnerable to gambling harms, such as younger adults, men, those who speak languages other than English and metropolitan residents. Altogether, the current study highlights the importance of reducing alcohol‐influenced gambling and its associated harms.

## 5 Conclusion

Using a representative sample of Victorians, this study showed that alcohol use while gambling is an important predictor of gambling‐related harms, even after accounting for gambling frequency, gambling expenditure and various socio‐demographic indicators. Furthermore, the combination of general HED and alcohol use while gambling was associated with substantially elevated harm, suggesting that individuals with heavier drinking patterns who also drink while gambling represent a particularly high‐risk group. While further research is warranted, the results demonstrate the importance of restricting alcohol availability and use in gambling situations and to consider demographic groups who are most vulnerable to reduce gambling harm.

## Author Contributions

All authors approved the final version and certify that their contributions meet the standards of the International Committee of Medical Journal Editors.

## Funding

The project was funded by the Victorian Responsible Gambling Foundation, now Victorian Department of Justice and Community Safety. Anne‐Marie Laslett was funded by the National Health and Medical Research Council (NHMRC GNT2016706). Heng Jiang was funded by the Australian Research Council Discovery Project (DP200101781).

## Conflicts of Interest

The authors declare no conflicts of interest.

## Supporting information


**Table S1:** Gambling related harms.
**Table S2:** Sensitivity analysis (without weights) of interaction effects of alcohol use while gambling (AWG) and heavy episodic drinking (HED) on the odds of any gambling harm (1 or 2+ harms indicated).

## Data Availability

The data used in this study are available from the Victorian Department of Justice and Community Safety upon reasonable request. The authors do not have permission to share the data publicly.
