# Supplementary material for: Alcohol Use Associated With Gambling Harm in a Population Representative Australian Sample
Source: Drug Alcohol Rev. 2026 May 4;45:e70164. doi: 10.1111/dar.70164 (PMC13139751; doi:10.1111/dar.70164)
Supplement: Supplementary file 1 — Table S1: Gambling related harms. Table S2: Sensitivity analysis (without weights) of interaction effects of alcohol use while gambling (AWG) and heavy episodic drinking (HED) on the odds of any gambling harm (1 or 2+ harms indicated). [file DAR-45-0-s001.docx]

| Table S1. Gambling related harms | |
| --- | --- |
| **Harm item - In the past 12 months, did any of these issues occur as a result of your gambling?** | Reported (%) |
| Reduction of your available spending money | 14.7% |
| Less spending on recreational expenses such as eating out, going to movies or other entertainment | 9.2% |
| Reduction of your savings | 12.8% |
| Sold personal items | 1.7% |
| Increased credit card debt | 1.7% |
| Had regrets that made you feel sorry about your gambling | 12.9% |
| Felt like a failure | 5.6% |
| Felt ashamed of your gambling | 7.3% |
| Felt distressed about your gambling | 6.6% |
| Spent less time with people you care about | 5.4% |
| Spent less on essential expenses such as medication, health care, and food^a^ | 2,9% |
| Experienced greater conflict in your relationships like arguing, fighting and ultimatums ^a^ | 3.5% |
| Been a victim of family or domestic violence ^a^ | 1.4% |
| Didn't attend fully to the needs of children ^a^ | 0.7% |
| *Note*.  ^a^Victoria-specific harm item |  |

| **Table S2.**  Sensitivity analysis (without weights) of interaction effects of alcohol use while gambling (AWG) and heavy episodic drinking (HED) on the odds of any gambling harm (1 or 2+ harms indicated). | | | | | | | | |
| --- | --- | --- | --- | --- | --- | --- | --- | --- |
|  | Any harm (0 vs. 1+) | | | | Number of harms | | | |
| Interaction alcohol while gambling × Heavy Episodic Drinking (HED) | Adjusted OR | 95% CI | | *p* | IRR | 95%CI | | p |
| Never × Never | 1.00 | - | - | - | 1.00 | - | - | - |
| Sometimes × Less than Monthly | 1.65 | 0.82 | 3.31 | .161 | **1.83** | **0.98** | **3.42** | **.048** |
| Sometimes × Monthly | 0.90 | 0.36 | 2.24 | .822 | 0.91 | 0.40 | 2.06 | .818 |
| Sometimes × Weekly or Daily | 1.79 | 0.43 | 7.39 | .420 | 3.76 | 0.92 | 15.45 | .065 |
| Often/Always × Less than Monthly | 2.02 | 0.58 | 7.06 | .270 | 1.52 | 0.46 | 4.98 | .494 |
| Often/Always × Monthly | 2.24 | 0.59 | 8.40 | .234 | 1.74 | 0.51 | 5.99 | .380 |
| Often/Always × Weekly or Daily | 3.39 | 0.68 | 16.94 | .137 | **6.20*** | **1.25** | **30.87** | **.026** |
| *Note.* OR=Odds Ration; IRR=Incidence Risk Ratio; ^a^ The models control for gambling frequency, gambling expenditure, and sociodemographic variables (see Table 1 for an overview); Exact p-values are provided for the multivariable analyses. | | | | | | | | |
